# How researchers frame scientific contributions to sustainable development: a typology based on grounded theory

**DOI:** 10.1007/s11625-016-0363-7

**Published:** 2016-04-18

**Authors:** Gabriela Wuelser, Christian Pohl

**Affiliations:** 1 Department of Environmental Systems Science, USYS TdLab, ETH Zurich, CHN K78, Universitaetstrasse 16, 8092 Zurich, Switzerland; 2 Network for Transdisciplinary Research (td-net), Swiss Academies of Arts and Sciences, P.O. Box, 3001, Bern, Switzerland

**Keywords:** Sustainability research, Project framing, Research design, Science–policy nexus, Grounded theory, Science studies

## Abstract

Given that research on sustainable development usually relates to real-world challenges, it requires researchers to align scientific knowledge production with concrete societal problem situations. To empirically explore how researchers frame scientific contributions when designing and planning projects, we conducted a qualitative study on land use–related projects based on the methodology of grounded theory. We identified major influence factors and various types of research design. Among the factors that influence project framing, scientific considerations were found to be more important than expected. Core characteristics of project framings concerned (a) type of scientific contributions envisaged; (b) real-world sustainability challenges addressed, and (c) researchers’ conceptions of how knowledge would reach its addressees. Three different types of project framing were found, suggesting that framing strongly depends on (the researchers’ perception of) how well a real-world problem situation is understood scientifically and how strongly are societal actors aware of the problem and act upon it. The spectrum of how researchers planned that knowledge would reach its addressees comprised communicating results to interactive conceptions allowing for mutual learning throughout the research process. The typology reveals a variety of useful and promising project framings for sustainable development research. The typology may serve to reconcile conceptual ideals and expectations with researchers’ realities.

## Introduction

Research for sustainable development is directed at triggering or supporting sustainability-oriented societal change. It serves to improve our knowledge and understanding for reconsidering the way we meet our needs. This usually implies aligning scientific knowledge production with societal problem situations, which requires going beyond disciplinary boundaries and the realm of science (Gallopin et al. 2001). A number of approaches are being explored to meet this extended demand, including (transformative) sustainability science (Kates et al. 2001; Komiyama and Takeuchi 2006), boundary work (Guston 2001), (open) knowledge systems (Cash et al. 2003; Tabara and Chabay 2013), integrative research (Van Kerkhoff 2013), and transdisciplinary research (Hirsch Hadorn et al. 2008; Jahn et al. 2012). These approaches are accompanied by studies that observe, structure, or critically reflect on research practice (Enengel et al. 2012; Miller 2013; Pohl et al. 2010; Van Kerkhoff and Szlezák 2010), for instance, to reveal factors of success or failure (Clark et al. 2011; Harris and Lyon 2013; Polk 2014; Van Kerkhoff and Szlezák 2010; Wiek et al. 2012) or to uncover research(er)s’ underlying normative assumptions (Couix and Hazard 2013; Lélé and Kurien 2011). However, the question of how researchers do master aligning research with real-world knowledge needs has not been addressed comprehensively so far.

The stage of designing projects and defining research questions crucially influences societal relevance of research, as will be elaborated on below. Therefore, we analyzed how researchers actually link scientific contributions to sustainable development, that is, how they orient the contents of their projects toward sustainability-related real-world problems *in the designing and planning stage*. The following were our two main research questions:

(a) Which factors influence project framing?

(b) Based on which characteristics can project framings be distinguished?

The insights we gained may serve as a heuristic, and inspire theoretical concepts and principles for project framing in sustainability research that are more strongly related to researchers’ realities.

Our analysis is structured as follows: First, we position our analysis in the current discussion on aligning scientific knowledge production with societal problem situations. After explaining our method, we present the patterns we found in researchers’ project framings. We then discuss the extent to which these patterns are congruent with, or add new insights to, the current understanding of research for sustainable development. We summarize our findings in a typology of how researchers frame scientific contributions to sustainable development.

## Framing research for sustainable development

When defined as “research that is directed at supporting sustainable development by providing knowledge about whether change is needed, and if so, how it can be brought about” (Wuelser et al. 2012, p. 82), research for sustainable development is conceived of as the process of generating scientific knowledge in conjunction with tackling sustainability challenges in the real world. In the literature, we found three issues being discussed with respect to making this connection when setting up and developing projects—a stage we refer to as *project or research framing*.

First, project framing is crucial because defining questions implies constraining possible answers. Project framing bears the risk of providing right answers to wrong questions (Kriebel et al. 2001). A research project, however, can provide insights only into those aspects of a sustainability issue that are analyzed. These aspects are selected in the stage of designing projects and defining research questions. Moreover, the manner in which a real-world sustainability challenge is perceived constrains or anticipates what is later regarded as a good solution to it (Rittel and Webber 1973). For research that aims at societal relevance, there have been calls for considering societal problem perceptions and knowledge requirements in the process of defining problems and research questions (Hirsch Hadorn et al. 2008).

Second, disciplinary perspectives are crucial. Science provides and advances understandings of phenomena in conformity with a discipline’s body of knowledge and conventions of good science (Fleck 1986). Disciplinary framings are not neutral to a societal sustainability challenge, as has, for instance, been shown in the case of tropical forest protection (Lélé and Kurien 2011) or alpine grassland restoration (Couix and Hazard 2013). Disciplinary framings reduce the complexity of real-world situations to the aspects relevant for the respective discipline, such as a natural area’s genetic diversity or economic value. Further reduction in complexity may come along with ideals of what would characterize a more sustainable situation, such as higher genetic diversity or higher earnings.

Third, connecting science to real-world challenges is crucial. This issue is discussed extensively in the literature in terms of how to organize the boundary between science and policy, that is, between “communities with different views of what constitutes reliable or useful knowledge” (Clark et al. 2011, p. 1). Aspects discussed in this context include communicating scientific results or integrating societal actors into knowledge co-production (Cash et al. 2006; Hirsch Hadorn et al. 2008; Krutli et al. 2010; Polk 2014; Sarewitz and Pielke Jr. 2007; Wuelser et al. 2012).

In this study, we integrate these aspects by empirically exploring researchers’ practice of project framing. The focus on researchers’ perspectives on project framing allowed us to stay open to all sorts of project-framing models, that is, to investigate whether, when, why, and how other people’s knowledge, perspectives, and positions influence this process, among other things. Therefore, we look at a whole range of sustainable development–related research. We do not limit ourselves to collaborative, participatory, or transdisciplinary projects (see Methods). Therefore, how researchers orient their projects in the initiatory stage to the societal concern of sustainable development is approached here by *describing what researchers think* should be studied, and why and how they conceive of the knowledge to be useful for handling a particular sustainability challenge.

## Methods

We used the qualitative methodology of grounded theory for analyzing problem framings as perceived by researchers—expressed in their own words—and to understand the underlying reasoning (Denzin and Lincoln 2005).

The full sample of projects comprised ten recent Swiss state-of-the-art research projects that, according to the proposals or personal communications, aimed explicitly to contribute to sustainable development. Projects related to land use issues and those in which the object of research or research context was a societally relevant sustainability challenge were selected. In line with the requirements of qualitative analysis, a heterogeneous sample of projects was compiled. The selected projects differed in terms of (a) the focus with respect to sustainability objectives, (b) disciplines involved (natural and social scientific), (c) form of research conducted (basic or applied; disciplinary, inter-, and transdisciplinary), (d) form of knowledge generated (systems, target, and transformation knowledge), (e) economic development context in which the research was conducted (industrialized and developing countries), and (f) project size (Table 1).

**Table 1:** Sample of research projects consisting of single Ph.D. studies, except for MOUNT (cluster project including ten Ph.D. studies in nine different research groups), BFUEL (consisting of two Ph.D. studies), and AQUA (consisting of four Ph.D. studies and a synthesis study)

Project (abbr.) (Number of interviews)	Project (short title)	Discipline/field	Country
POLL (2)	Ecosystem service pollination	Ecology	India
PALM (1)	Oil palm expansion	(Applied) Ecology	Indonesia
LIV (1)	Forest and livelihoods	Forestry and development	Madagascar
BFUEL (3)	Biofuel crop production: debates and impacts	Sociology and human geography	Ethiopia
MOUNT (2)	Land use in mountain regions (MOUNTLAND)	Several natural and social science fields	Switzerland
FOR (2)	Impact of drought on forest development	(Forest) Ecology	Switzerland
WAT (2)	Water-related environmental services	Physical geography	Kenya/Tanzania
AQUA (3)	Water stress and management options	Human and physical geography	Switzerland
CARB (2)	Carbon sequestration potential	Ecosystem sciences	Panama
LEG (1)	Crop-livestock systems	Plant nutrition	Nicaragua

We collected data by semi-structured interviews (Flick 2002) and content analysis of research proposals. Following theoretical sampling (Corbin and Strauss 2008), a set of 12 full interviews with PhD students, post-docs, and senior scientists was conducted, complemented later by four shorter interviews with supervising professors. The main criterion for selecting the interview participants was their involvement in setting up the projects, because these persons were assumed to know the most about the reasons for choosing the respective project designs. At the time of the interviews, several projects were ongoing, while others had just been finished. All interviews were recorded and transcribed.

To mutually relate, compare, and adjust data collection, analysis, summarizing paraphrases, and interpretation, these tasks were conducted iteratively and recursively, as suggested in the methodology of grounded theory. This allowed us to not only thoroughly study the individual cases while being open to all sorts of characteristics that distinguish project framings but also to develop the respective characterizing categories during the course of the study (Corbin and Strauss 2008; Glaser and Strauss 1967).

## Results

The results presented below were obtained using six showcase projects that typify the range of project framings identified (Table 2). For the sake of clarity, the characterizing categories represent ideal–typical simplifications (Weber 1962) of what are mostly smooth transitions in reality.

**Table 2:** Summarizing paraphrases of core characteristics describing how investigated research projects framed their contributions to sustainable development

Project (abbr.)	Influence factors in research project development	Type of scientific contribution	Sustainability challenge addressed	Conception of how knowledge will reach addressees
POLL	State of research and scientific discussion Personal research interests Available knowledge on local realities due to previous work and existing contacts	Clarify whether coffee crop productivity in Kodagu, India, depends on bee pollination facilitated by natural forest fragments in a highly diverse landscape	Potential future disappearance of native forest fragments and resultant biodiversity loss, as well as potentially decreasing crop yields	Contribution of new insights to scientific discussion Dissemination of knowledge to local coffee farmers through interactions during research and follow-up workshop
BFUEL	Personal interests Funder requirements (double project) Adaptations due to initiation of a broader research project	Describe the impacts of biofuel crop production on local livelihoods and the debate of the underlying values and world views	Strongly diverging political positions and opinions on harms and benefits of biofuel crop production	Contribution of basic understanding to follow-up research Publication of results in target group language
MOUNT	Running scientific experiments and existing infrastructure Funder requirements (interdisciplinarity) Personal interests Developing a novel approach Expertise of consortium	Model landscape development trends in terms of future ecosystem service provision in Swiss mountain regions due to climate and land use change, and resulting socio-economic consequences Develop political steering mechanisms for dealing with these expected changes	Impacts of probable future climate change on sensitive regional mountain ecosystems and potentially negative land use trends	Anchoring of the research in the case study regions and sensitizing local actors through regular stakeholder dialogues throughout the research
WAT	Preceding study concerning the problem Institutional context: long-term research strategy of group Available contextual knowledge Shortcoming of approach	Model and spatially visualize the availability and distribution options of water-related ecosystem services in the Pangani Basin, Kenya and Tanzania, under present and different future conditions	Increasing regional water scarcity with resulting conflicts against the background of increasing population pressure and anticipated impacts of climate change	Stakeholder workshops for triggering debate among different water users and for discussing development options with them (Regular) contact with the crucial regional actor (water authority) during the research upon its identification
CARB	Knowledge gap Thirst for knowledge Existing infrastructure and afforestation experiment Obvious knowledge demand	Quantify plant productivity and carbon sequestration potentials of a traditional pasture and of an afforestation plot in tropical ecosystems using the example of Panama	Global climate change	Policy advice allowing better informed decisions The authority in charge of specifying an emissions-trading scheme based on afforestation was interested in the results but not in exchanging ideas during the research A follow-up research project taking up the results to develop concrete options for action was envisaged
LEG	State of research Expertise of research group Existing partnership with local applied research institution Knowledge demand Access to knowledge of local realities	Determine the use options and performance of the legume Canavalia in terms of nutrient budgets, soil fertility, and agricultural productivity at plot level for introduction into traditional smallholder crop-livestock systems of the Nicaraguan hillsides	Decreasing productivity of smallholder systems in rural Nicaraguan hillsides leading to poverty and soil depletion due to agricultural intensification and population pressure	Good contact with farmers of the project’s Central American (applied) research partner allowed close collaboration with the farmers from the beginning The research was to be conducted on-farm and the farmers’ knowledge, needs, and experiences were integrated

### Factors influencing research project development

**Fig. 1:**
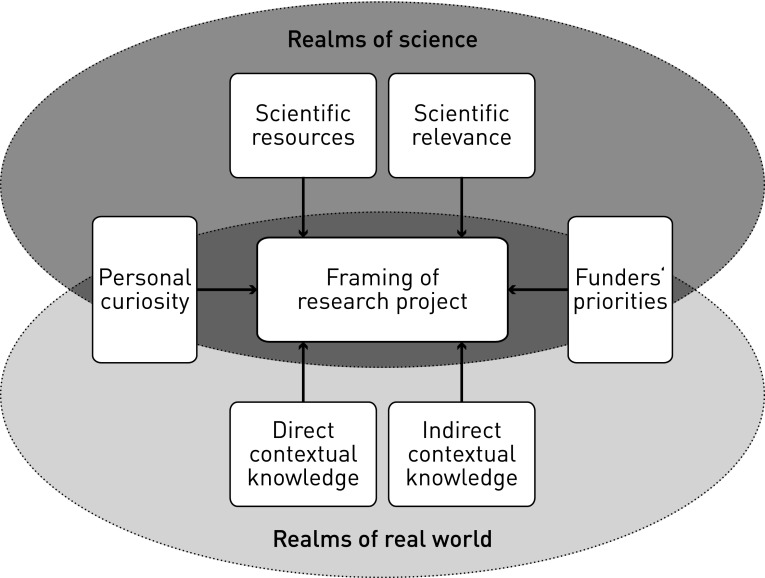
Influences in early stage of framing research for sustainable development can ideal-typically be attributed to scientific or real worlds. Note that these simplified distinctions are made for the sake of clarity. In reality, the identified influence factors are not necessarily independent of each other: Personal curiosity may arise out of direct contextual knowledge, and scientific relevance may influence funders’ priorities, for example

Most of the factors influencing project development were revealed from the interviewees’ accounts of how project ideas arose. These factors were found to be either of a scientific nature or of a practical nature, that is, inspired by a real-world problem situation (Fig. 1).

*Scientific influence factors* *(1)* are the available scientific resources as well as the scientific relevance attributed to certain topics and methods. Both scientific resources and relevance are related mostly to researchers’ own specialized fields. The exception was the project MOUNT, which was explicitly being constructed as an interconnected, interdisciplinary endeavor, thus involving repeated discussions in the stage of project framing on the disciplines or fields to include.

*Scientific resources* *(1a)* encompass current state of knowledge, methods and approaches of the field, and methodological competences and infrastructure available to the project team. The simulation models available, for instance, allow researchers to consider certain system parameters but hinder them from considering others:

“The other thing is the methodical limitation. There are people in the project team who bring in certain strengths. (…) The starting point was these models. We wanted the natural scientific models to generate outputs that can be taken up by the socioeconomic ecosystem services models; thus, this translation of landscape development into ecosystem services. This was the real aim. That is why we needed to concentrate strongly on these models and their capabilities. So, the parameters that were easy to work out or were feasible [in these models] were the first priority ecosystem services we covered. Water, for instance, is one that up until now has not been integrated in the landscape development model. Although we are working on it, it is simply not there yet (translated from MOUNT 1 p. 6,7).”

Research infrastructure can be a decisive factor when it is required for experimental methods, especially when it is costly. One of the projects reused measurement towers installed for an earlier project: “It was a coincidence that we simply had these contacts. But that’s how science often works and that the [measurement] towers were already there; because the installation costs are, of course, enormous” (translated from CARB 2, p. 3).

*Scientific relevance* *(1b)* encompasses taking up actual scientific discussions or trends in the field in terms of questions studied and methodologies used, as well as introducing or advancing innovative concepts and approaches. POLL, for instance, aimed at substantiating scientific discussion on biodiversity conservation and ecosystem services:

“At the time we wrote the proposal, there was (…) some controversy about this idea. Many people accept that a good argument for conservation, for preserving natural areas, is the pollination services they supply to farmers. If farmers can be persuaded that you need natural areas to raise crop production through the pollination services that those natural areas provide, then they might indeed be persuaded to keep some patches of natural forest. (…) We were interested to see whether the arguments that theoreticians and ecologists were putting forward can actually be applied to that context (POLL 2, p. 1).”

Advancing scientific approaches, for instance, encompasses linking different disciplines on the basis of modeling: A feedback loop would be closed by simulating how suggested policy measures—derived by ecological modeling, among other things—impact ecosystems (Huber et al. 2013). Extending the ecosystem services approach by incorporating the value attributed by different groups of society to certain services as a function of access and time is another example of how advancing scientific approaches influences project framing (WAT 1, p. 4).

*Problem*-*related influence factors* *(2)* are based on researchers’ direct and indirect contextual knowledge of real-world situations.

*Direct contextual knowledge* *(2a)* includes what researchers know and have learned about a sustainability challenge, its history, current trends, and local perceptions, typically through fieldwork. In some projects, researchers were familiar with such local realities because of previous research of their own or that of team members in the same region or socio-cultural context. These projects were embedded in a longer term presence of the research group in the region, featuring a broader strategy oriented to support societal problem solving.

*Indirect contextual knowledge* *(2b)* is knowledge that researchers access via networks and project partners. LEG, for instance, benefited from collaboration with a national applied research institution working in the area, in which it had cultivated long-term contacts. This local research partner provided the researchers with access to farmers who placed trust in them so that they could consider local needs, views, priorities, and knowledge: “We are not the experts in the field. Neither the Ph.D. student nor myself could tell a priori which kind of research these farmers needed. We are only able to do so thanks to our partners (LEG, p. 6).”

*Further influence factors* *(3)* includes personal curiosity of the researchers and the priorities set by research funders. Whether such influences are motivated by scientific advancement, societal problem solving, or represent a combination of the two depends on the respective researchers’ priorities.

*Personal curiosity* *(3a)* is motivated by scientific understanding, for instance, the wish to better understand or model an observed phenomenon or development.

*Funders’ priorities* *(3b)* and requirements were also found to be influential in some projects. In the case of MOUNT, a crucial funder demanded, for example, connecting experimental work with socio-economic and landscape modeling, as well as with a political study, to trigger interdisciplinary integration (MOUNT 1, p. 1).

### Characteristics of project framings

We found that the characteristics of researchers’ project framings differed in terms of (1) the types of intended scientific contributions; (2) real-world sustainability challenges addressed, and (3) researchers’ conceptions of how knowledge would reach its addressees.

#### Types of scientific contributions

Although the projects were from different disciplines and concerned a thematically diverse set of sustainability challenges, they featured, in essence, three types of scientific contributions: Researchers envisaged providing an improved (fundamental) understanding of certain phenomena, outlining (more) sustainable resource use patterns, and determining selected parameters in specific contexts.

*Providing improved (fundamental) understanding of certain phenomena (1a)* is a common type of research. It involves identifying cause–effect relationships or deepening the understanding of behavior patterns and their drivers. If directed at sustainable development, it extends the knowledge base, which may eventually inform judgments and decisions regarding the ways we meet our needs. POLL, for example, investigated cause–effect relationships in testing whether coffee crop production in a certain area depended significantly on pollination services facilitated by nearby high-biodiversity areas (Boreux et al. 2013). Clarifying worldviews, social positions, and self-understanding in the biofuel debate is another example:

“I would like to contribute to seeing what underlies these positions—why do some have this interest and others have another one—to clarify whether there are needs that have not been considered thus far, or why someone has taken up a certain stance over this issue. Moreover, to be able to assess in this terms of pros and cons—what is really important and which positions are fed by self-interest” (translated from BFUEL 2, p. 15).

*Outlining (more) sustainable resource use patterns (1b)* builds on scientifically well-described phenomena. Different disciplinary knowledge bases are integrated to develop a bigger picture. In the investigated land use research, such bigger pictures consisted of regional resource use patterns. The projects provide knowledge on respective steering and governance options. This was framed, for instance, as the impact of global change and political framing conditions on future development of dominant land use forms in Swiss mountain regions (MOUNT 1, p. 3), and as water distribution options in the Pangani Basin in Kenya and Tanzania in consideration of economic development, livelihood, and ecosystem needs (Notter 2010).

*Determining selected parameters in specific contexts (1c)* builds on generally well-described phenomena but provides or deepens their understanding for a specific (local) context. For instance, an established micrometeorological measurement method was used to determine the carbon sequestration potentials of two predominant land use types in the tropics (Wolf et al. 2011), resulting in the first dataset of this parameter for this climatic zone. As another example, local soils best suited for introducing a selected legume species into a traditional crop-livestock system were identified to increase on-farm productivity and soil fertility (Douxchamps et al. 2010). The research built on well-described nutrient cycle mechanisms.

#### Sustainability challenge addressed

For analyzing the sustainability challenges the research projects referred to, the descriptions of the challenges were emphasized, specifically, the most distinctive characteristics of these descriptions. The problems’ state of being manifest, recognized, and resolved in the real-world turned out to be a fundamental feature. The problems are differentiated as those in an early stage of being identified by people and those that are widely recognized with rather clear strategies to be acted upon—virtually representing two ends of a spectrum (For a detailed analysis of researchers’ sustainability conceptions, see Wuelser 2014).

*Problems that are about to be identified (2a)* in the real world are possible, anticipated negative consequences of current or past practices. They are not yet or only just apparent. In the eyes of the researchers, people are not necessarily aware of them nor do they argue about whether or what about the current development is problematic, and for whom. There might be no consensus on what the problem is. Consequently, coping strategies are hardly discussed, or they are not broadly approved. Projects featuring these characteristics addressed potential negative consequences of land use practices as well as those of external influences on land use, such as climate and global change. One project referred to the potential future problem of insufficient bee pollination supposedly leading to decreasing productivity in a coffee-growing area due to diminishing bee habitats in form of natural forest areas. It addressed the tendency to turn these natural habitats into monoculture plantations. The coffee farmers were reported to be largely unaware of this potential problem because it had not (yet) become apparent to them (POLL 1, p. 14). In the case of BFUEL, it was contested whether positive or negative consequences of biofuel crop production prevailed, that is, what was about to become problematic, and for whom:

“I saw that the topic was extremely controversial and that you can find virtually every possible statement on what biofuels do or don’t do. Really, every one of them is possible, depending considerably on the actor or the actor group, whether this is described in a very positive, very negative, or rather balanced way” (BFUEL 2, p. 3).

*Widely recognized problems featuring rather clear strategies to be acted upon (2b)* are described as being rather obvious and well known, as well as uncontested in principle. The process of identifying strategies for mitigating such problems seems to be ongoing or partly even advanced. The problem of water scarcity in the Pangani Basin in Kenya and Tanzania, for example, had been recognized and fought by international development organizations for decades. Problem-solving efforts were made on different levels, but different sectoral policies conflicted with each other. The advocated strategy for acting upon the problem—introducing a water rate—had so far been unsuccessful, as the researchers assumed, because the responsible authority had too little power (WAT 1, pp. 15/16).

#### Researchers’ conceptions of how knowledge reaches its addressees

The researchers’ project descriptions and explanations mostly entailed specific notions of how the knowledge they produced would reach its addresses. They can be sorted into two ideal typical categories: Disseminating results and interactive concepts.

*Disseminating results* *(3a)* means passing on scientific knowledge in a unidirectional manner to selected actors within or outside academia. This comprises, but is not limited to, publishing in scientific journals or other suitable, typically target-group-specific media. In so doing, contributing to societal change is handled mostly separately from the actual research process. It can be conceptualized both as forms of one-time communication activities after research is finalized and as (repeated) communication activities during research. Depending on the situation, the knowledge is considered to be of direct use for acting upon a sustainability challenge, or it flows into intermediary projects or activities, that is, it is supposed to be of use indirectly. Researchers’ intent to communicate the results upon publication directly to the national authority in charge of climate politics—referred to as policy brief—(CARB 2, p. 2) exemplifies the one-time communication model. Repeated communication activities comprise, for instance, using a number of overall stakeholder meetings throughout the research “to convey the idea of implementing the political measures that one will suggest, also a little outwards and—besides the scientific aim—to some degree also to pursue the goal of having an impact on the real politics” (MOUNT 1, p. 4).

*Interactive concepts of how knowledge reaches its addressees (3b)* are also referred to as mutual learning processes (Klein et al. 2001). They are common in transdisciplinary and most participatory research approaches and ideally form an integral part of the actual research. This means that expertise, priorities, and questions of non-academic actors are considered or even integrated into the ongoing research and regarded as relevant in substance. The interactive concepts we encountered were built on existing and well-working contact networks providing access to local people’s needs and knowledge, which considerably facilitated close collaboration with them. Using a bottom-up participatory process, the local farmers, for instance, selected the—most promising from their perspective—crop species to study, which also considerably increased trust in the research, as well as its direct usefulness on the ground (LEG, p. 3).

## Discussion

### Project framing is based on researchers’ scientific competences

Our analysis suggests that when it comes to framing concrete project ideas, research for sustainable development is predominantly influenced by scientific considerations (Table 2). Projects are framed based on the perspective of researchers’ scientific competences rather than being based on societal actors’ problem perceptions. The stories told about how a project idea emerged mostly started with existing scientific resources, contacts and interests, previous research to follow up, or specific funding opportunities to seize. Real-world problem–related considerations were mentioned later and to stress the relevance of the research. Societal actors’ problem perspectives and knowledge needs influenced research questions predominantly indirectly. Project framings seemed to be more strongly shaped by the type of research objects previously analyzed, known methods, measuring devices acquired, established working relations, and the intention to advance methods and theories, while the wish to support society in handling the sustainability problem was given more of an overarching importance or it built the context of application.

This finding is somewhat in contrast to the literature claiming that to be relevant for handling sustainability challenges, research should start from societal actors’ problem perceptions and knowledge needs, ideally using joint problem framing or at least framing projects from a real-world perspective (Hirsch Hadorn et al. 2008; Jahn et al. 2012; Wuelser et al. 2012). While the significance of scientific considerations in project set up is absolutely necessary and understandable, research is after all a scientific endeavor, it is also clear that including non-academic actors and stakeholders potentially increases the relevance of research. This may not only happen through applying transdisciplinary approaches to the project but also through contacts, exchanges, collaborations, or fieldwork prior to the project. Furthermore, our results suggest that the problem orientation of research might be hindered by (a) the dominant incentives and rewards of the scientific system, such as pressure to publish as much as possible and in high-ranking journals or funding schemes focusing on disciplinary excellence only and (b) the fact that while such claims sound simple in theory, fulfilling them is very demanding, laborious, and time-consuming.

### State of understanding matters

**Fig. 2:**
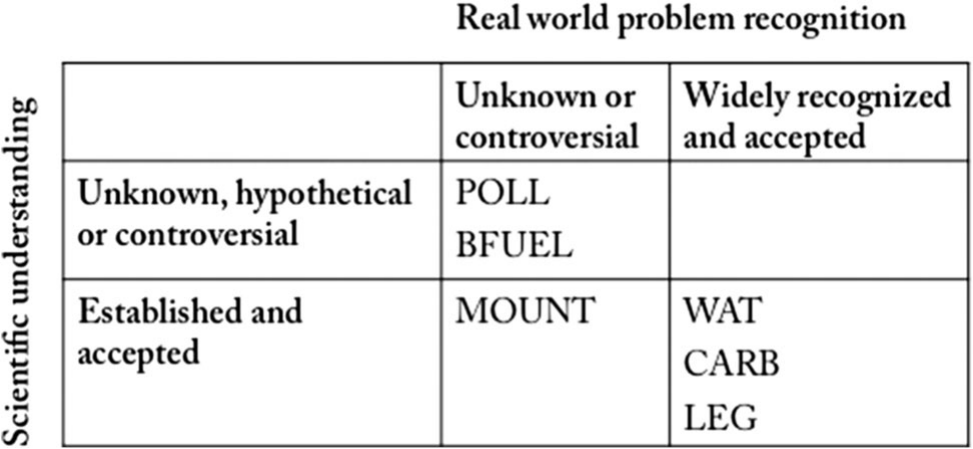
Project framing is influenced by both state of scientific understanding of phenomena related to a problem and problem recognition by relevant actors and stakeholders in real world

In line with Pohl et al. (2010), Clark et al. (2011), and Enengel et al. (2012), we find not one best, but heterogeneous patterns of how projects envisage contributing to sustainable development. Furthermore, our study points to how strongly the state of understanding, both in science and society, influences problem framing (Fig. 2).

In our sample, the types of scientific contributions to sustainable development, as determined in the stage of project framing, depended on the scientific understanding of the phenomena being considered. The questions that can be explored are constrained by the scientific understanding of the phenomena that influence, explain, or cause a sustainability challenge. In the context of producing knowledge for tackling sustainability problems, the state of scientific understanding on the one hand precludes certain research questions to be pursued. BFUEL, for instance, was started at a time when the impact of biofuel on local livelihoods was largely unknown. The controversy about whether—and under what circumstances—biofuel is a blessing or a curse was hardly understood scientifically (BFUEL 1, p. 7). Scientifically very well described mechanisms, on the other hand, allow for the formulation of straighter applicable sets of research questions with respect to problem solving. If the problem is broadly recognized by the crucial stakeholders as well, the project design can be well tailored for acting upon the problem straightforwardly. However, this may happen at the expense of scientific rewards in form of high-ranking publications (e.g. LEG, p. 6). As these examples show, the influences of scientific understanding and societal problem recognition on project framing seem to be interconnected (Fig. 2). Recognition of a problem by societal actors and decision makers mirrors, to some extent, the process of finding a way to act upon it. However, the choice of research questions seems to be influenced by a combination of the extent to which a problem has become manifest, the extent to which it is recognized by societal actors and stakeholders, including the degree of consensus about what is problematic, and the existence of a strategy to act upon the problem.

### Interactive ways of knowledge exchange do not necessarily mean greater relevance

In our sample, researchers’ conceptions of how knowledge would reach its addressees partly followed the linear (deficit) model of knowledge dissemination (Van de Kerkhof and Wieczorek 2005). The projects using interactive concepts revealed that—in some contrast to the literature (e.g. Hirsch Hadorn et al. 2008; Klein et al. 2001; Wiek et al. 2012)—interactive ways of knowledge exchange do not *generally* represent a more suitable way of contributing to societal problem solving. These models may be less effective in case (a) scientific understanding and societal problem recognition are very poor, so researchers have to first develop a scientific understanding of the phenomenon. Also in some cases, (b) researchers are familiar with the real-world problem situation and scientific understanding is sufficient, mutual learning interactions with stakeholders yield little added value for both sides. Note that in the latter case, there may instead be a specific linear knowledge demand from stakeholders to researchers, similar to selecting the most promising crop species in the case of LEG.

Whether scientific findings find their way into policy making during or after the research may not be that crucial. Our expectation that researchers following the linear model typically communicate results at the end of research directly to the addressees turned out to be too simplistic, however. Some researchers planned to communicate their scientific findings at several stages of the research process. Further, it is worth stressing that the majority of the researchers envisioned their knowledge contribution to become effective via multi-level procedures, for example, being taken up by a follow-up or a larger research, development, or other sort of intermediary project.

### Project framing typology

The ways in which the researchers framed their projects can, in summary, be classified along the three types of scientific contributions described above (Table 3):

**Table 3:**
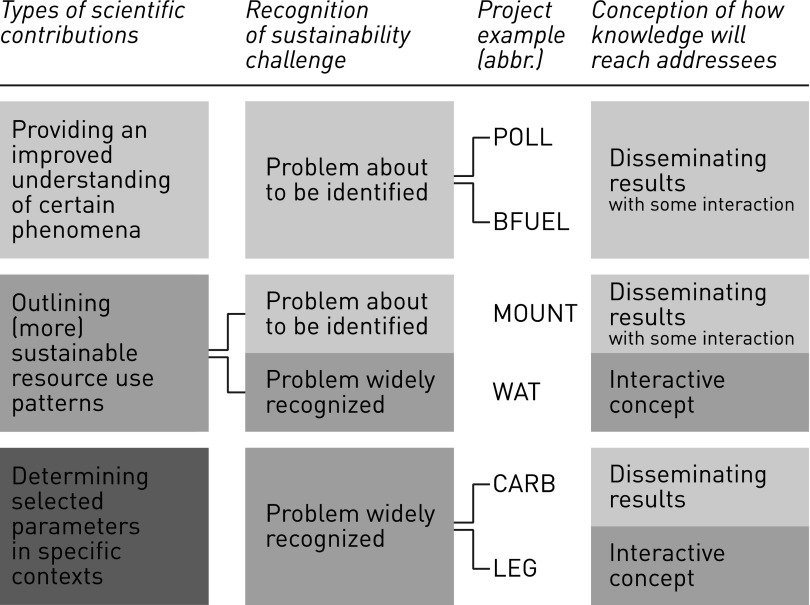
Three models of how researchers frame scientific contributions to sustainable development

- The first type of project framing aims at providing a more fundamental or differentiated understanding of the crucial aspects of a (potentially) problematic development in the real world. Researchers deem the sustainability challenge to be inadequately understood scientifically and insufficiently recognized: Societal actors are perceived to be hardly aware of the problem or they disagree on what the problem actually is. The scientific understanding and societal recognition of the sustainability challenge feature major open questions and are characterized by controversies about what should be deemed problematic. Such project framing is also applied when the state of knowledge fails to satisfactorily explain certain observations.
- The second type of project framing provides—in the case of land use issues—knowledge about the bigger picture of resource management and its longer term effects. By outlining more sustainable resource use patterns in the form of scenarios, researchers aim to provide a basis for political debates and decision-making. The precondition for such simulation work is reliable scientific understanding of the underlying phenomena. However, in our sample, recognition of the sustainability challenge to which these projects were related, differed. This implies that scenarios might be useful contributions at different stages in societal problem solving, ranging from early discussions on what trends may be problematic to advanced shaping of details.
- The third type of project framing refers to real-world sustainability challenges that are broadly recognized and little contested. Such projects provide contextually specified knowledge, answering particular questions or knowledge demands for enabling societal actors to take action in some way or implement a policy. Scientifically, the research builds on well-understood phenomena.

With respect to how knowledge is to reach its addressees, different approaches are selected. They seem not to depend on the state of scientific or societal problem understanding and are thus not assigned to the three types of project framing.

## Conclusions

In the context of sustainable development, it is essential to try and maximize the potential societal relevance of scientific contributions in the early stage of designing and planning projects. Our analysis of researchers’ considerations during project framing provides the following insights into this process:

- In contrast to claims found in the literature, project framing in research for sustainable development is predominantly influenced by scientific considerations, such as existing scientific infrastructure, previous research to follow-up, or specific funding opportunities to seize. Real-world problem–related considerations are of minor significance and are often mentioned to stress the relevance of the research. Moreover, research problem orientation might be hindered by the dominant incentives and rewards of the scientific system, such as pressure to publish as much as possible and in high-ranking journals or funding schemes focusing on disciplinary excellence only. In this respect, research for sustainable development seems to not differ considerably from other types of research. Taking the problem-related influence factors more seriously, for instance, by involving relevant non-academic knowledge-holders in project framing, might be central to changing this situation.
- The major consideration of researchers during project framing can be summarized in three models. They correspond to the types of envisaged scientific contributions: (1) Providing an improved understanding of certain phenomena, (2) outlining (more) sustainable resource use patterns, and (3) determining selected parameters in specific contexts (Table 3). The typology points out that considering both the extent to which a problem is scientifically understood and the process of how societal problems are recognized and tackled seem to be crucial for conceiving policy-relevant knowledge contributions to sustainable development. Considering these two factors and their implications for sustainability research might be worthwhile to allow for a diversity of research models.

The typology sheds light on how researchers frame scientific contributions to sustainable development. In addition to describing researchers’ reasoning, the typology can be used to improve project design and reconcile sustainability science expectations with research realities. It is a starting point for reflecting on one’s own or a team’s assumptions with respect to a sustainability problem, its understanding in science and society, and the knowledge required to act upon it. Through such reflection, the typology supports researchers by characterizing more precisely what they do, why, and how so that they can try to maximize the potential societal relevance of their work.

The typology is based on a sample of ten Swiss-based land use projects, and it may be extended to different types of sustainability issues. Therefore, we do not claim it is complete. Nevertheless, we hope these findings help to advance policy-relevant research for sustainable development by bridging the “discrepancy between what people need and want when they live in an area, and what researchers are happy to investigate” (POLL 1, p. 21).
